# Self-limiting paratransgenesis

**DOI:** 10.1371/journal.pntd.0008542

**Published:** 2020-08-18

**Authors:** Wei Huang, Sibao Wang, Marcelo Jacobs-Lorena

**Affiliations:** 1 Department of Molecular Microbiology and Immunology, Malaria Research Institute, Johns Hopkins Bloomberg School of Public Health, Baltimore, Maryland, United States of America; 2 CAS Key Laboratory of Insect Developmental and Evolutionary Biology, Institute of Plant Physiology and Ecology, Shanghai Institutes for Biological Sciences, Chinese Academy of Sciences, Shanghai, China; National Institute of Allergy and Infectious Diseases, UNITED STATES

## Abstract

Presently, the principal tools to combat malaria are restricted to killing the parasite in infected people and killing the mosquito vector to thwart transmission. While successful, these approaches are losing effectiveness in view of parasite resistance to drugs and mosquito resistance to insecticides. Clearly, new approaches to fight this deadly disease need to be developed. Recently, one such approach–engineering mosquito resident bacteria to secrete anti-parasite compounds–has proven in the laboratory to be highly effective. However, implementation of this strategy requires approval from regulators as it involves introduction of recombinant bacteria into the field. A frequent argument by regulators is that if something unexpectedly goes wrong after release, there must be a recall mechanism. This report addresses this concern. Previously we have shown that a *Serratia* bacterium isolated from a mosquito ovary is able to spread through mosquito populations and is amenable to be engineered to secrete anti-plasmodial compounds. We have introduced a plasmid into this bacterium that carries a fluorescent protein gene and show that when cultured in the laboratory, the plasmid is completely lost in about 130 bacterial generations. Importantly, when these bacteria were introduced into mosquitoes, the bacteria were transmitted from one generation to the next, but the plasmid was lost after three mosquito generations, rendering the bacteria non-recombinant (wild type). Furthermore, no evidence was obtained for horizontal transfer of the plasmid to other bacteria either in culture or in the mosquito. Prior to release, it is imperative to demonstrate that the genes that thwart parasite development in the mosquito are safe to the environment. This report describes a methodology to safely achieve this goal, utilizing transient expression from a plasmid that is gradually lost, returning the bacterium to wild type status.

## Author summary

The intolerable burden of malaria demands the development of novel approaches to fight this deadly disease. Previously, the engineered symbiotic bacterium *Serratia* AS1 was shown in the laboratory to be able to spread through mosquito populations and render mosquitoes resistant to the parasite. However, a major concern of regulators for the release of such engineered bacteria into the field is that there is no option for “recall” in case something goes wrong. Here we show that *Serratia* AS1 loses plasmids as it replicates in mosquitoes and in culture, reverting to wild type. We also show that horizontal transfer of the plasmid from *Serratia* AS1 to other bacteria is extremely rare, as this was undetectable in the mosquito and in culture. Our results suggest that initial field trials can make use of this reversible system whereby released recombinant bacteria expressing anti-plasmodial compounds from a plasmid revert to wild type at a predictable rate.

## Introduction

While global malaria cases and deaths declined dramatically over the past decades, malaria incidence since 2014 has remained at similar levels [1]. Clearly, implementation of additional measures to contain this deadly disease are urgently needed. The mosquito microbiota shows great potential for fighting malaria parasite transmission by the mosquito, as the most vulnerable stages of parasite development occur in the mosquito gut, a compartment shared with the microbiota. Moreover, during parasite development within the blood bolus, the number of the surrounding bacteria increases by several hundred-fold [2]. We are exploring a strategy (paratransgenesis) that consists of engineering mosquito symbiotic bacteria to deliver anti-pathogen effector molecules [2–4]. We have shown that the *Serratia* AS1 bacterium strain isolated from *Anopheles* ovaries, can spread into mosquito populations and that this bacterium can be engineered to express anti-parasite genes [3]. Mosquitoes that carry these bacteria are largely refractory to the parasite. Translation of these findings to the field will require the approval of regulatory agencies. However, a major concern of regulators is that there should be an option for “recall” in case something goes wrong. This report addresses this concern.

## Methods

### Ethics statement

All animal experimental procedures were reviewed and approved by the Johns Hopkins University Animal Care and Use Committee (ACUC) under Protocol Number: M018H18.

#### Mosquito rearing

*Anopheles stephensi* (Dutch strain) [5] mosquitoes were maintained on 10% sterile sucrose at 27°C and 80 ± 5% relative humidity under a 14 h/10 h day-night cycle. Larvae were fed on cat food pellets and ground fish food supplement.

#### Bacterial culture and introduction into mosquitoes via sugar meal

*Escherichia coli* DH5α (Invitrogen) was used for DNA cloning and plasmid amplification. *Serratia* AS1 and *E*. *coli* were cultured in LB broth or on agar plates at 28°C and 37°C, respectively. Bacteria were harvested by centrifugation (3,000xg, 10 min), washed twice in sterile phosphate buffered saline (PBS), and resuspended in 5% (wt/vol) sterile sucrose solution to obtain 10^7^ cells/ml. The bacterial suspension was added to sterile cotton pads and provided to two-day old mosquitoes for 24 h, then bacterial cotton pads were replaced with new sterile cotton pads containing 5% sucrose solution.

#### Bacterial genomic DNA isolation

Genomic *Serratia* AS1 DNA was extracted from pelleted cultures using a Qiagen DNeasy Blood and Tissue kit (cat. 69504).

#### *Serratia* AS1 sensitivity to antibiotics

*Serratia* AS1 was cultured overnight at 200 rpm at 28 ^o^C and 10^4^ cells were spread on LB plates with or without ampicillin, kanamycin, chloramphenicol, streptomycin, apramycin, neomycin, tetracycline, rifampin, metronidazole, 5-fluorocytosine, spectinomycin and carbenicillin, and cultured for 24 h or 48 h at 28 ^o^C.

#### Construction of vectors

The apramycin cassette was amplified from the pDB47 plasmid [2] using primers ApraF and ApraR (S7 Table), PnptII-eGFP was amplified from the pUT-mini-km2-GFP [3] plasmid using primers GFPF and GFPR, with *Not*I recognition sites incorporated at the 5'- and 3'-ends. The PCR product was fused into the *Not*I site of the plasmid pUT-mini-Tn5-Cm [6] using the In-Fusion HD Cloning Kit, to generate pUT-mini-Apra-GFP. The *nptII* promoter was amplified from the pUT-mini-km2-GFP plasmid using primers pnptII1 and pnptII [2], mCherry was amplified from the mCherry-*Serratia* genome [3] using primers mCherry3 and mCherry4. The pnptII-mCherry fragment was amplified from the *nptII* promoter and mCherry fragments by fusion-PCR (Primer: pnptII1 and mCherry4). To construct pHL662-mCherry, pnptII-mCherry was PCR-amplified using primers mCherry5 and mCherry6, the PCR product was digested with *Xma*I and *Hind*III and cloned into the *Xma*I and *Hind*III sites of the plasmid pHL662 [7]. To construct punc-119c-mCherry, pnptII-mCherry was amplified using primers mCherry7 and mCherry8 and the PCR product was digested with *Spe*I and cloned into the *SpeI* site of the plasmid punc-119c [8].

#### Measurement of plasmid loss rate

The pMyc Vec2 [9], punc-119c, pet-GFP [10], pHL662, SK(SK-YFP-ST1-A) [11], and pDB47 plasmids were separately electro-transformed into *Serratia* AS1. *Serratia* with or without the plasmid was cultured in liquid LB with or without antibiotic. The culture was started as 10^−3^ or 10^−6^ OD, and samples were tested for concentration and plasmid loss every 4 or 8 h (log phase) [12]. Generation = log2(Final concentration/initial concentration). Plasmid loss in the mosquito used *Serratia* that had apramycin-resistance (Apra^R^) and GFP genes integrated into its genome. Two-day-old adult mosquitoes (50 males and 50 females) were allowed to feed for 24 h on a cotton pad moistened with 5% sterile sucrose solution containing 10^7^ *Serratia* /ml carrying the punc-119c-mCherry or pHL662-mCherry plasmids. Larvae and adult mosquitoes of different stages were homogenized and plated on LB plates with or without apramycin for the determination of fluorescent colony numbers.

#### Plasmid copy number (PCN) determination

DNA of *Serratia* AS1 was extracted from pelleted cultures using a Qiagen DNeasy Blood and Tissue kit (cat. 69504). The PCN was calculated by dividing the copy number of the plasmid kanamycin gene by the single copy chromosomal gene LuxS (Accession number: AP019009.1) as control. Applied Biosystems Step One Plus Real-time PCR System was used for qPCR amplification and detection. Real-time qPCR reactions were performed in triplicate using 20 μl mixtures. Each reaction contained 10 μl of 2× SYBR Green PCR Master Mix (Toyobo, Kyoto, Japan), 1 μl of template DNA, and 12.5 pmol of each primer. The PCR reaction was conducted for all amplicons with the following cycling conditions: 2 min at 95 ^o^C, followed by 40 cycles of 95 ^o^C for 15 s, 60 ^o^C for 20 s, and 72 ^o^C for 30 s. Upon completion of 40 cycles of PCR amplification, a dissociation step of ramping the temperature from 55 ^o^C to 95 ^o^C steady for 20 min was performed, while the fluorescence signal was continually monitored for melting curve analysis. We determined the cycle threshold (Ct) values after automatic adjustment of the baseline and manual adjustment of the threshold using Stepone software V2.3. The plasmid DNA standard curve was established according to the method of Lee et al [13]. Briefly, the standard curve includes a plot of the Ct values versus the log concentration of the plasmid DNA standard. For total DNA sample, the absolute quantity of both plasmid and chromosomal DNA were obtained by interpolating the Ct value against the standard curve.

#### Plasmid horizontal transfer in culture

*E*. *coli* was selected for its known ability to acquire plasmids and for use in conjugation positive controls. In addition, *P*. *agglomerans* was selected because it is one of the most common, abundant and well-studied mosquito symbionts. Donor and recipient cells were cultured separately overnight at 200 rpm at 28 ^o^C (for *Serratia AS1* and *P*. *agglomerans*) or 37 ^o^C (for *E*. *coli*). We used two methods to measure the horizontal gene transfer. For the first method (solid medium; Table 2), 10^12^ donor and recipient cells (ratio = 1:1) were mixed in 50 ml PBS, spread on ten 15 cm-LB agar plates and incubated for 6 h at 28 ^o^C, after which all bacteria were collected from the plates with 100 ml LB. For the second method (static medium; S4 Table), 10^12^ donor and recipient cells (ratio = 1:1) were mixed in 200 ml LB culture and incubated for 6 h at 28 ^o^C without shaking [14, 15]. For both methods, the entire culture was mixed with soft agar containing kanamycin and apramycin (or kanamycin and ampicillin for the *E*. *coli* controls), and plated on 15 cm-LB agar plates (20 ml each plate) (S1 Fig).

#### Horizontal transfer in mosquitoes

Two-day old mosquitoes were fed on *Serratia* (with chromosomally integrated apramycin and GFP genes) carrying the punc-119c-mcherry or pHL662-mcherry plasmids. Larvae or adult mosquito midguts of different stages were homogenized and spread on kanamycin LB plates for counting of fluorescent colonies. Horizontal transfer rate = mCherry CFUs/Total CFUs.

#### Statistical analyses

Multiple-sample comparisons were analyzed using the non-parametric Kruskal-Wallis test and medians were compared using Dunn’s test. Other statistical significance was calculated using Student’s t-test for unpaired comparisons between two treatments. A value of *P* < 0.05 was considered to be statistically significant. All statistical analyses were performed using GraphPad Prism version 7.00 for Windows (GraphPad Software).

## Results

**Fig 1:**
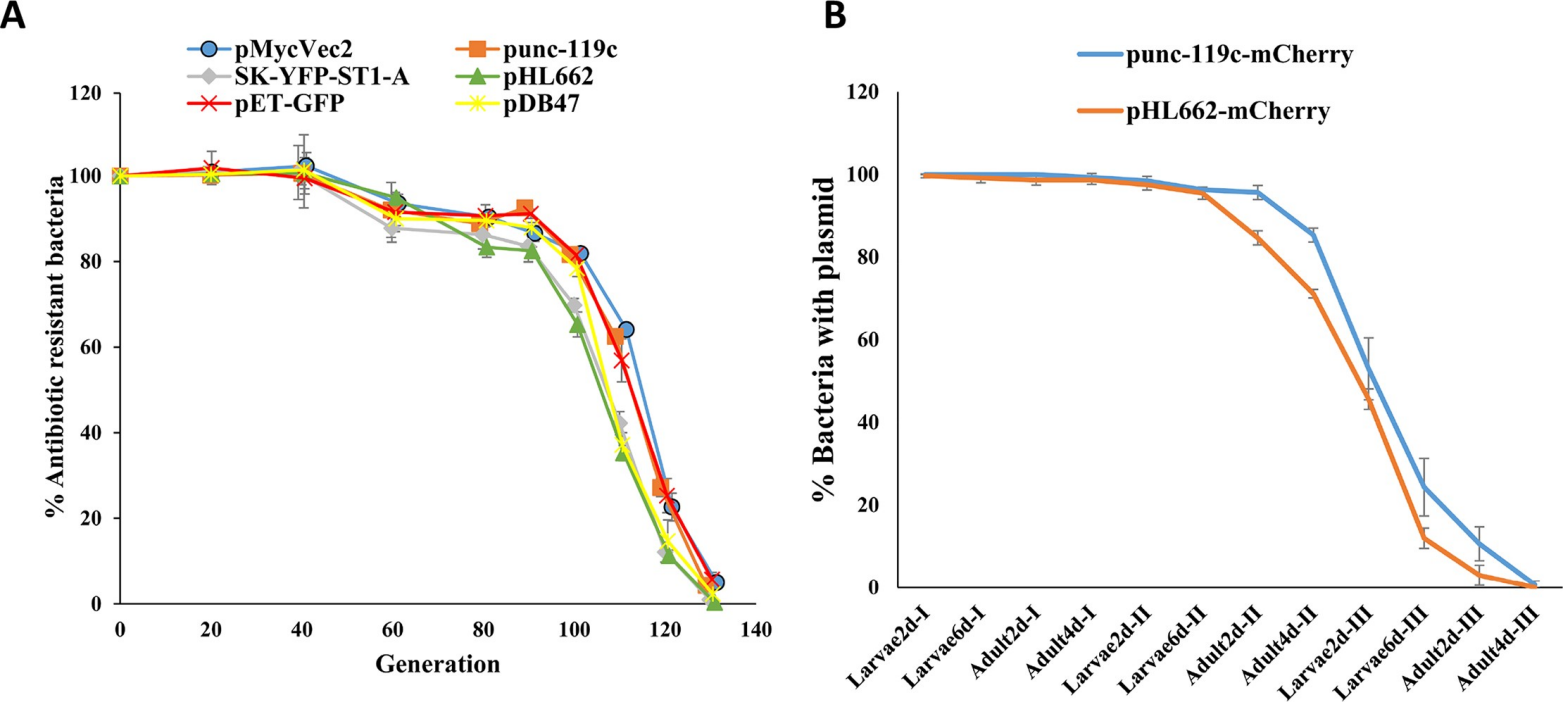
Measurement of plasmid loss rate. **A,** *Serratia* AS1 carrying plasmids encoding antibiotic resistance (S2 Table) were grown in liquid culture. Samples were taken every 4 or 8 h and colony forming units (CFU) were determined on LB plates with and without antibiotics, to measure bacteria growth and plasmid loss events. Data from three independent experiments were pooled. **B,** Chromosomally GFP-labelled (green) and apramycin-resistant *Serratia* AS1 carrying the indicated mCherry-tagged (red) plasmid were fed to 100 mosquitoes (50 males and 50 females) and these mosquitoes were propagated through three generations (I–III). Larvae (2- and 6-day old) and adults (2- and 4-day old) were homogenized and plated on apramycin plates to determine what percent of the green-fluorescent bacteria carry the mCherry-tagged plasmid. Data from three independent experiments were pooled.

We investigated whether upon replication, *Serratia* AS1 would lose an introduced plasmid, reverting to wild type. This *Serratia* AS1 is sensitive to apramycin, kanamycin, streptomycin, rifampin and spectinomycin (S1 Table). We tested six plasmids carrying different origins of replication plus an antibiotic-resistance gene (S2 Table). In culture, all plasmids were lost in about 130 generations (Fig 1A). There was a lag in loss of antibiotic resistance during the first ~100 generations because each bacterium carried ~15 or ~38 copies of the plasmid (S3 Table) and antibiotic resistance is lost only with the complete loss of the plasmid. There was a small but significant difference in the plasmid loss rate (*P*<0.01): pHL662, SK(SK-YFP-ST1-A), and pDB47 plasmids were lost faster than the pMycVec2, punc-119c and pet-GFP plasmids (Fig 1A). We chose one plasmid from each group to investigate the loss rate when the bacteria are maintained through multiple mosquito generations (Fig 1B) [3]. To distinguish *Serratia* AS1 from the natural mosquito microbiome components, we stably integrated the GFP (green) and apramycin-resistance genes into its genome and introduced mCherry-tagged (red) plasmids into this bacterium. These bacterial colonies display both green and red fluorescence. However, if the plasmid gets lost, the colonies will display only green fluorescence. We found that the plasmids were completely lost in three mosquito generations, and pHL662-mCherry was lost faster than punc-119c-mCherry (*P*<0.05) (Fig 1B).

An additional potential concern is whether the genetic material carried by the plasmids will horizontally transfer to neighboring bacteria. We fed mosquitoes with *Serratia* AS1 that had chromosomally-integrated a GFP gene (green) and that carry either the punc-119c-mcherry-kanR or the pHL662-mcherry-kanR plasmids (red) and followed these bacteria for three mosquito generations. Horizontal transfer to microbiome bacteria would result in red (but not green) fluorescent kanamycin-resistant bacteria, while the donor bacteria have green or both green and red fluorescence. A total of 648,860 (punc-119c-mCherry) and 861,250 (pHL662-mCherry) bacteria were assayed and no evidence for horizontal transfer was obtained (Table 1, S5 and S6 Tables). Because the number of bacteria that can be assayed in mosquito experiments is limited, we designed horizontal transfer experiments in culture that used a much larger bacteria number (10^12^) (S1 Fig). No transfer was detected from *Serratia* AS1 to *Escherichia coli* or to *Pantoea agglomerans*, the latter being a common component of mosquito microbiomes (Table 2 and S4 Table). In a positive control, the transfer rate from *E*. *coli S17-1* with an F^+^ plasmid to *Serratia* AS1 was 4.3×10^−6^ (solid plate; Table 2) or 4.3×10^−6^ (static medium; S4 Table). In short, horizontal transfer from *Serratia* AS1 to bacteria from the mosquito microbiome or to bacteria in culture was undetectable.

**Table 1:** Horizontal transfer in mosquitoes.

Plasmid	LB (total bacteria number)	LB/Kan GFP^+^ & mCherry^+^ (donor bacteria number)	LB/Kan mCherry^+^-only (recipient bacteria number)
**punc-119c-mCherry**	648,860	18,419	0
**pHL662-mCherry**	861,250	15,357	0

*In vivo* horizontal transfer of the punc-119c-mcherry and pHL662-mcherry plasmids from *Serratia* AS1 to the mosquito microbiome. *Serratia* carrying the plasmid were fed to mosquitoes (50 males and 50 females) and maintained for three generations (S5 and S6 Tables). No evidence of plasmid transfer was detected.

**Table 2:** Horizontal transfer in culture (solid plate).

Donor cell (10^12^)	Carried plasmid	Recipient cell (10^12^)	Transfer rate
***Serratia* AS1**	punc-119c	*Serratia* AS1/Apra+GFP	0
***Serratia* AS1**	punc-119c	*P*. *agglomerans/*Apra+GFP	0
***Serratia* AS1**	punc-119c	*E*. *coli* (DH5α)/Apra+GFP	0
***Serratia* AS1**	pHL662	*Serratia* AS1/Apra+GFP	0
***Serratia* AS1**	pHL662	*P*. *agglomerans*/Apra+GFP	0
***Serratia* AS1**	pHL662	*E*. *coli* (DH5α)/Apra+GFP	0
***E*. *coli* (S17-1)**	F^+^	*Serratia* AS1	7.8×10^−6^
***E*. *coli* (S17-1)**	pHL662 (F^-^)	*Serratia* AS1	0

Horizontal transfer of the indicated plasmid was performed using 10^12^ donor and recipient bacteria each, incubated on solid plates for 6 h (Method 1; see Methods). Positive and negative control experiments were performed using *E*. *coli(S17-1)* carrying an F^+^ or the pHL662 (F^-^) plasmid, respectively. See also S1 Fig and S4 Table. The punc-119c and pHL662 plasmids carry a kanamycin-resistance gene; the F^+^plasmid carries an ampicillin-resistance gene; *Serratia* AS1 is naturally resistant to ampicillin. Transfer rate = transconjugants/recipients. Pooled data from two independent experiments.

## Discussion

The low genetic stability and ease of manipulation of plasmid-based overexpression are advantageous features for initial field experiments, as they minimize environmental risk. Bacteria that do not inherit a plasmid may have a fitness advantage in that they multiply faster. In addition, plasmid loss rate as bacteria multiply depends on plasmid rate of replication that in turn determines Plasmid Copy Number per bacterium (PCN) [16,17]. In support of this assertion, plasmid pHL662 that has a PCN 14.6 is lost faster (Fig 1) than plasmid punc-119c that has a PCN 37.8 (S3 Table). Therefore, the half-life of recombinant gene expression after bacteria release may be adjusted to some extent.

Whereas *Serratia* AS1 loses the plasmid relatively rapidly during logarithmic growth in culture (about 130 generations or 40~45 hours), the dynamics in the mosquito are very different. Growth of bacteria in the mosquito is limited by nutrient conditions, oxygen levels and other micro-environment factors, amounting to a much slower replication. *Serratia* AS1 persists in mosquitoes for three generations (50~60 days). Importantly, about 50% of these bacteria carry the plasmid at the beginning of the F3 generation, implying that these bacteria express the anti-parasite effectors. Recall that the intent here is to obtain evidence for safety of the expressed effector molecules, not to implement a broad anti-malaria campaign.

The finding that no plasmid horizontal transfer was detected in mosquitoes has two caveats. One is that it is not known whether transfer may have occurred to non-culturable mosquito bacteria, as such an assay is not easy to implement. The other is that the total number of bacteria that can be tested in an *in vivo* experiment is relatively small. To address this caveat, we assayed horizontal transfer in culture from *Serratia* AS1 to *E*. *coli* and *P*. *agglomerans*, a ubiquitous component of the mosquito microbiome using very high bacteria numbers (10^12^) and conditions that favor bacteria-bacteria contact. Even under these conditions that strongly favor plasmid transfer, none was observed. Whereas the possibility of a very rare gene transfer in the mosquito cannot be excluded, such event should have minimal consequences as the genes encoded by the plasmid (fluorescent proteins, anti-plasmodial small proteins) would not confer fitness advantage to the recipient cell.

The approach proposed here is envisioned as a prelude for full implementation of the paratransgenesis strategy. Once environmental safety of the anti-*Plasmodium* compounds is corroborated, *Serratia* carrying effector genes stably integrated in their chromosome would be constructed. Introduction into the field can be accomplished by mixing the bacteria with attractive sugar baits (both male and female mosquitoes need sugar for survival [18]). Because the engineered *Serratia AS1* is sexually transmitted from male to female mosquitoes and is passed from one generation to the next by attaching to eggs, it can efficiently spread into mosquito populations [3]. Paratransgenesis is compatible with other malaria-containment measures, including the use of insecticides and mosquito transgenesis.

## Supporting information

S1 Table *Serratia* AS1 sensitivity to antibiotics. *Serratia* AS1 was plated on LB plates with or without 50 μg/ml ampicillin, 50 μg/ml kanamycin, 34 μg/ml chloramphenicol, 50 μg/ml streptomycin, 80 μg/ml apramycin, 10 μg/ml tetracycline, 10 μg/ml rifampin, 50 μg/ml metronidazole, 0.5 μg/ml 5-fluorocytosine or 50 μg/ml spectinomycin and cultured for 24 h or 48 h at 28 ^o^C.
(DOCX)

S2 Table Plasmids used in this study. (DOCX)

S3 Table Plasmid copy number in *Serratia* AS1. Plasmid copy number was determined by quantitative PCR as detailed in Methods. Pooled data from three independent experiments.
(DOCX)

S4 Table Horizontal transfer in culture (in static medium). Horizontal transfer of the indicated plasmid was performed using 10^12^ donor and recipient bacteria each, incubated in liquid medium without agitation for 6 h (Method 2; see Methods). Positive and negative control experiments were performed using *E*. *coli(S17-1)* carrying an F^+^ or the pHL662 (F^-^) plasmid, respectively. See also S1 Fig and Table 2. The punc-119c and pHL662 plasmids carry a kanamycin-resistance gene; the F^+^.plasmid carries an ampicillin-resistance gene; *Serratia* AS1 is naturally resistant to ampicillin. Transfer rate = transconjugants/recipients. Pooled data from three independent experiments.
(DOCX)

S5 Table punc-119c-mCherry horizontal transfer in mosquitoes. Chromosomally GFP-labelled *Serratia* AS1 carrying the indicated mCherry-tagged plasmid were fed to mosquitoes and these mosquitoes were propagated through three generations (I–III). Larvae (2- and 6-day old) and adults (2- and 4-day old) were homogenized and plated on LB plates without (Total bacteria) and with kanamycin to determine percent fluorescent bacteria carrying the plasmid. The *Serratia* AS1 fed to the mosquitoes form green- and red-fluorescent kanamycin-resistant colonies, while transfer of the plasmid to bacteria from the mosquito microbiome would form red-fluorescent-only kanamycin-resistant colonies. No such colonies were found. Pooled data from three independent experiments.
(DOCX)

S6 Table pHL662-mCherry horizontal transfer in mosquitoes. Chromosomally GFP-labelled *Serratia* AS1 carrying the indicated mCherry-tagged plasmid were fed to mosquitoes and these mosquitoes were propagated through three generations (I–III). Larvae (2- and 6-day old) and adults (2- and 4-day old) were homogenized and plated on LB plates without (Total bacteria) and with kanamycin to determine percent fluorescent bacteria carrying the plasmid. The *Serratia* AS1 fed to the mosquitoes form green- and red-fluorescent kanamycin-resistant colonies, while transfer of the plasmid to bacteria from the mosquito microbiome would form red-fluorescent-only kanamycin-resistant colonies. No such colonies were found. Pooled data from three independent experiments.
(DOCX)

S7 Table Oligonucleotide primers used in this study. (DOCX)

S1 Fig Horizontal transfer in culture. Rate of transfer of plasmid between donor and recipient strains. Only bacteria that received the plasmid can grow on [apramycin + kanamycin] LB plates.
(TIF)
