# Supplementary material for: Self-limiting paratransgenesis
Source: PLoS Negl Trop Dis. 2020 Aug 18;14(8):e0008542. doi: 10.1371/journal.pntd.0008542 (PMC7454989; doi:10.1371/journal.pntd.0008542)
Supplement: S1 Table — Serratia AS1 was plated on LB plates with or without 50 μg/ml ampicillin, 50 μg/ml kanamycin, 34 μg/ml chloramphenicol, 50 μg/ml streptomycin, 80 μg/ml apramycin, 10 μg/ml tetracycline, 10 μg/ml rifampin, 50 μg/ml metronidazole, 0.5 μg/ml 5-fluorocytosine or 50 μg/ml spectinomycin and cultured for 24 h or 48 h at 28 oC. (DOCX) [file pntd.0008542.s001.docx]

**S1 Table. *Serratia* AS1 sensitivity to antibiotics**

| **Antibiotic** | | **Resistant** | | **Antibiotic** | | **Resistant** | |
| --- | --- | --- | --- | --- | --- | --- | --- |
| Ampicillin | | yes | | Tetracycline | | no | |
| Kanamycin | | no | | Rifampin | | no | |
| Chloramphenicol | | yes | | Metronidazole | | yes | |
| Streptomycin | | no | | 5-Fluorocytosine | | yes | |
| Apramycin | | no | | Spectinomycin | | no | |

*Serratia* AS1 was plated on LB plates with or without 50 µg/ml ampicillin, 50 µg/ml kanamycin, 34 µg/ml chloramphenicol, 50 µg/ml streptomycin, 80 µg/ml apramycin, 10 µg/ml tetracycline, 10 µg/ml rifampin, 50 µg/ml metronidazole, 0.5 µg/ml 5-fluorocytosine or 50 µg/ml spectinomycin and cultured for 24 h or 48 h at 28 ^o^C.
