# Supplementary material for: Self-limiting paratransgenesis
Source: PLoS Negl Trop Dis. 2020 Aug 18;14(8):e0008542. doi: 10.1371/journal.pntd.0008542 (PMC7454989; doi:10.1371/journal.pntd.0008542)
Supplement: S2 Table — (DOCX) [file pntd.0008542.s002.docx]

**S2 Table. Plasmids used in this study**

| **Plasmid** | **Source** | **Resistance** | **Origin of replication** |
| --- | --- | --- | --- |
| pHL662 | Addgene | Kanamycin | p15a origin |
| SK-YFP-ST1-A | Addgene | Kanamycin | pSC101 origin |
| punc-119c | Addgene | Kanamycin | R6K origin |
| pMycVec2 | Addgene | Kanamycin | ColE1 origin |
| pET-GFP | Addgene | Kanamycin | pBR322 Origin |
| pDB47 | Wang, 2012^3^ | Apramycin | ColE1 origin |
