# Supplementary material for: Self-limiting paratransgenesis
Source: PLoS Negl Trop Dis. 2020 Aug 18;14(8):e0008542. doi: 10.1371/journal.pntd.0008542 (PMC7454989; doi:10.1371/journal.pntd.0008542)
Supplement: S3 Table — Plasmid copy number was determined by quantitative PCR as detailed in Methods. Pooled data from three independent experiments. (DOCX) [file pntd.0008542.s003.docx]

**S3 Table. Plasmid copy number in *Serratia* AS1**

| **Plasmid** | **Copy number** |
| --- | --- |
| pHL662-mCherry | 14.6±7.0 |
| punc-119c-mCherry | 37.8±12.2 |

Plasmid copy number was determined by quantitative PCR as detailed in Methods. Pooled data from three independent experiments.
