# Supplementary material for: Self-limiting paratransgenesis
Source: PLoS Negl Trop Dis. 2020 Aug 18;14(8):e0008542. doi: 10.1371/journal.pntd.0008542 (PMC7454989; doi:10.1371/journal.pntd.0008542)
Supplement: S4 Table — Horizontal transfer of the indicated plasmid was performed using 1012 donor and recipient bacteria each, incubated in liquid medium without agitation for 6 h (Method 2; see Methods). Positive and negative control experiments were performed using E. coli(S17-1) carrying an F+ or the pHL662 (F-) plasmid, respectively. See also S1 Fig and Table 2. The punc-119c and pHL662 plasmids carry a kanamycin-resistance gene; the F+.plasmid carries an ampicillin-resistance gene; Serratia AS1 is naturally resistant to ampicillin. Transfer rate = transconjugants/recipients. Pooled data from three independent experiments. (DOCX) [file pntd.0008542.s004.docx]

**S4 Table. Horizontal transfer in culture (in static medium)**

| Donor cell (10^12^) | Carried plasmid | Recipient cell (10^12^) | Transfer rate |
| --- | --- | --- | --- |
| *Serratia* AS1 | punc-119c | *Serratia* AS1/Apra+GFP | 0 |
| *Serratia* AS1 | punc-119c | *P. agglomerans/*Apra+GFP | 0 |
| *Serratia* AS1 | punc-119c | *E. coli* (DH5α)/Apra+GFP | 0 |
| *Serratia* AS1 | pHL662 | *Serratia* AS1/Apra+GFP | 0 |
| *Serratia* AS1 | pHL662 | *P. agglomerans/*Apra+GFP | 0 |
| *Serratia* AS1 | pHL662 | *E. coli* (DH5α)/Apra+GFP | 0 |
| *E. coli* (S17-1) | F^+^ | *Serratia* AS1 | 4.3×10^-6^ |
| *E. coli* (S17-1) | pHL662 (F^-^) | *Serratia* AS1 | 0 |

Horizontal transfer of the indicated plasmid was performed using 10^12^ donor and recipient bacteria each, incubated in liquid medium without agitation for 6 h (Method 2; see Methods). Positive and negative control experiments were performed using *E. coli(S17-1)* carrying an F^+^ or the pHL662 (F^-^) plasmid, respectively. See also S1 Fig and Table 2. The punc-119c and pHL662 plasmids carry a kanamycin-resistance gene; the F^+^.plasmid carries an ampicillin-resistance gene; *Serratia* AS1 is naturally resistant to ampicillin. Transfer rate = transconjugants/recipients. Pooled data from three independent experiments.
