# Supplementary material for: Self-limiting paratransgenesis
Source: PLoS Negl Trop Dis. 2020 Aug 18;14(8):e0008542. doi: 10.1371/journal.pntd.0008542 (PMC7454989; doi:10.1371/journal.pntd.0008542)
Supplement: S6 Table — Chromosomally GFP-labelled Serratia AS1 carrying the indicated mCherry-tagged plasmid were fed to mosquitoes and these mosquitoes were propagated through three generations (I–III). Larvae (2- and 6-day old) and adults (2- and 4-day old) were homogenized and plated on LB plates without (Total bacteria) and with kanamycin to determine percent fluorescent bacteria carrying the plasmid. The Serratia AS1 fed to the mosquitoes form green- and red-fluorescent kanamycin-resistant colonies, while transfer of the plasmid to bacteria from the mosquito microbiome would form red-fluorescent-only kanamycin-resistant colonies. No such colonies were found. Pooled data from three independent experiments. (DOCX) [file pntd.0008542.s006.docx]

**S6 Table. pHL662-mCherry horizontal transfer in mosquitoes**

| **Stage** | **Total bacteria** | **Kan+GFP+mCherry** | **Kan+mCherry only** |
| --- | --- | --- | --- |
| L2-I | 16,130 | 2,063 | 0 |
| L6-I | 20,060 | 1,625 | 0 |
| A2-I | 14,810 | 1,481 | 0 |
| A4-I | 23,740 | 4,935 | 0 |
| L2-II | 21,190 | 863 | 0 |
| L6-II | 18,190 | 713 | 0 |
| A2-II | 14,630 | 628 | 0 |
| A4-II | 198,100 | 2,438 | 0 |
| L2-III | 159,400 | 603 | 0 |
| L6-III | 219,400 | 8 | 0 |
| A2-III | 155,600 | 0 | 0 |
| A4-III |  |  |  |
| **Total** | **861,250** | **15357** | **0** |

Chromosomally GFP-labelled *Serratia* AS1 carrying the indicated mCherry-tagged plasmid were fed to mosquitoes and these mosquitoes were propagated through three generations (I – III). Larvae (2- and 6-day old) and adults (2- and 4-day old) were homogenized and plated on LB plates without (Total bacteria) and with kanamycin to determine percent fluorescent bacteria carrying the plasmid. The *Serratia* AS1 fed to the mosquitoes form green- and red-fluorescent kanamycin-resistant colonies, while transfer of the plasmid to bacteria from the mosquito microbiome would form red-fluorescent-only kanamycin-resistant colonies. No such colonies were found. Pooled data from three independent experiments.
