# Supplementary material for: Self-limiting paratransgenesis
Source: PLoS Negl Trop Dis. 2020 Aug 18;14(8):e0008542. doi: 10.1371/journal.pntd.0008542 (PMC7454989; doi:10.1371/journal.pntd.0008542)
Supplement: S7 Table — (DOCX) [file pntd.0008542.s007.docx]

**S7 Table. Oligonucleotide primers used in this study.**

| **Primer** | **Sequence (5’- 3’)** | **Notes** | |  |
| --- | --- | --- | --- | --- |
| Punc119cL1 | CAAATCCGTGACCTCGACAC | | For Punc-119c plasmid qPCR | |
| Punc119cR1 | GCGCAAATCGATATCGGACA | |  |  |
| pHL662L1 | TTCGTCTTCACCTCGAGTCC | | For pHL662 plasmid qPCR | |
| pHL662R1 | TGAAGCGCATGAACTCCTTG | |  |  |
| LuxSL1 | CCCTGAGCTGAACGAGTACC | | For single copy gene qPCR in chromosome | |
| LuxSR1 | CGCCGTTATCCAGAATGTGC | |  |  |
| ApraF | GCCTAGGCCGCGGCCGCATGCCCTC  GTGGTCAGGTCT | | For construction of pUT-mini-Apra-GFP | |
| ApraR | AGTGCTTGCGGCAGCGTGCGTAGCTCTTGATCCGGCAAAC | |  |  |
| GFPF | GCCTAGGCCGCGGCCGCATGCCCTCGTGGTCAGGTCT | |  |  |
| GFPR | GCCTAGGCCGCGGCCGCATGCCCTCGTGGTCAGGTCT | |  |  |
| pnptII1 | GCACGCTGCCGCAAGCACTC | | For pnptII promoter amplification | |
| pnptII2 | TACACAAGTGCGGCCGCCTACTTGTACAGCTCGTCCA | |  |  |
| mCherry3 | TCGCCCTTGCTCACCATCTTCCCAACCTTACCAGAG | | For mCherry promoter amplification | |
| mCherry4 | CTACTTGTACAGCTCGTCCAT | |  |  |
| mCherry5 | CCCCCCGGGGCACGCTGCCGCAAGCACTC | | For construction of pHL662-pnptII-mCherry | |
| mCherry6 | CCCAAGCTTCTACTTGTACAGCTCGTCCAT | |  |  |
| mCherry7 | GGACTAGTGCACGCTGCCGCAAGCACTC | | For punc-119c-pnptII-  mCherry | |
| mCherry8 | GGACTAGTCTACTTGTACAGCTCGTCCAT | |  |  |
