# Supplementary figures and images for: Self-limiting paratransgenesis
Source: PLoS Negl Trop Dis. 2020 Aug 18;14(8):e0008542. doi: 10.1371/journal.pntd.0008542 (PMC7454989; doi:10.1371/journal.pntd.0008542)

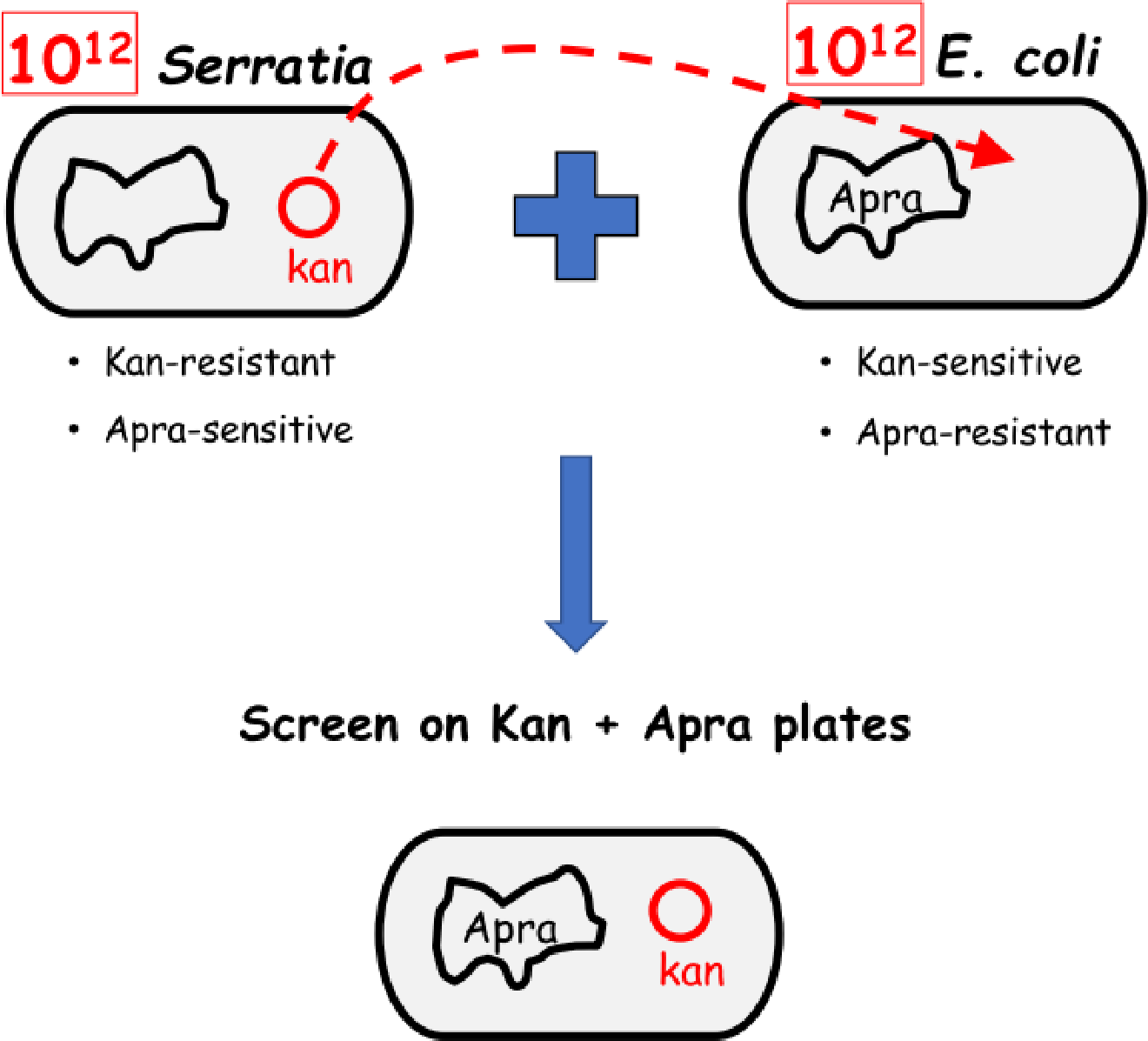

Supplement: S1 Fig — Rate of transfer of plasmid between donor and recipient strains. Only bacteria that received the plasmid can grow on [apramycin + kanamycin] LB plates. (TIF) [file pntd.0008542.s008.tif]
